# Reply to the correspondence on salivary biomarkers in breast cancer diagnosis: A systematic review and diagnostic meta‐analysis

**DOI:** 10.1002/cam4.6352

**Published:** 2023-09-27

**Authors:** Maryam Koopaie, Mahnaz Fatahzadeh, Sajad Kolahdooz

**Affiliations:** ^1^ Tehran University of Medical Sciences Tehran Iran; ^2^ Department of Diagnostic Sciences, Rutgers School of Dental Medicine Newark New Jersey USA; ^3^ USERN Tehran University of Medical Sciences Tehran Iran

First, we would like to thank the Dehdari Ebrahimi et al. for their detailed review of our study. Firstly, as we know, there is a narrow line between systematic review and review studies. One of the common limitations in systematic reviews is abundance of details from which an acceptable and valid conclusion cannot be deduced. Regarding your first point, “the eligibility criteria used in the study do not provide enough details about which salivary biomarkers were investigated,” I would like to draw your attention to 2.2 on page 2646 where, we feel, adequate information in this regard has been provided. If you mean that sufficient explanation about the type of selected biomarker was not given, we must remind you that all salivary biomarkers in saliva have been investigated for breast cancer diagnosis; however, due to limitations of the articles, the selection may have been incomplete. Nevertheless, more analyses was performed based on the classification in the subgroup analysis.

In regard to the actual biomarkers studied (e.g., c‐erbB‐2, vascular endothelial growth factor, epidermal growth factor, etc.), one of our aims during the study design was to perform meta‐regression. In our study, there were five articles related to c‐erbB‐2; however, the confusion matrix could not be inferred from these articles even after follow‐up emails (further details of the evidence are available) and these studies excluded from our meta‐analysis. Therefore, meta‐regression was not possible with this approach.

As meta‐regression is not recommended for samples less than three studies. Therefore, meta‐regression was not performed in this view. Additional information on the number of studies for each salivary biomarker in breast cancer detection together with classification of biomarkers may help further understanding. These are provided in a review study by Koopaie et al.^1^

Second, we agree that pooling data on different indicator tests (i.e., salivary biomarkers) in a meta‐analysis of diagnostic test accuracy studies, if not accompanied by further subgroup analyses, are prone to bias and reader confusion. Therefore, we would like to draw the attention of the esteemed readers to Table S2, in which the subgroup analysis according to the following six criteria (mean age of patients, saliva type, biomarker measurement method, sample size, type of control, and nations) was performed. Subgroup analyses depend on statistical power, so it usually makes no sense to conduct it when the number of studies is small,^2^ and it is a bad idea to use aggregate study information in subgroup analyses because this may introduce ecological bias.^3^ Given the above limitations, authors found it most logical to perform the subgroup analysis based on these six subgroups.

Thirdly, with respect to publication bias heterogeneity, we agree that funnel plots often show a tendency for smaller rather than large studies to report different and often more inflated effect sizes. Therefore, as per recommendation of Cochrane manual for systematic review of the diagnostic test accuracy, we used Deeks method as seen in Figure 1, which is also in line with ours.^4^

**FIGURE 1:**
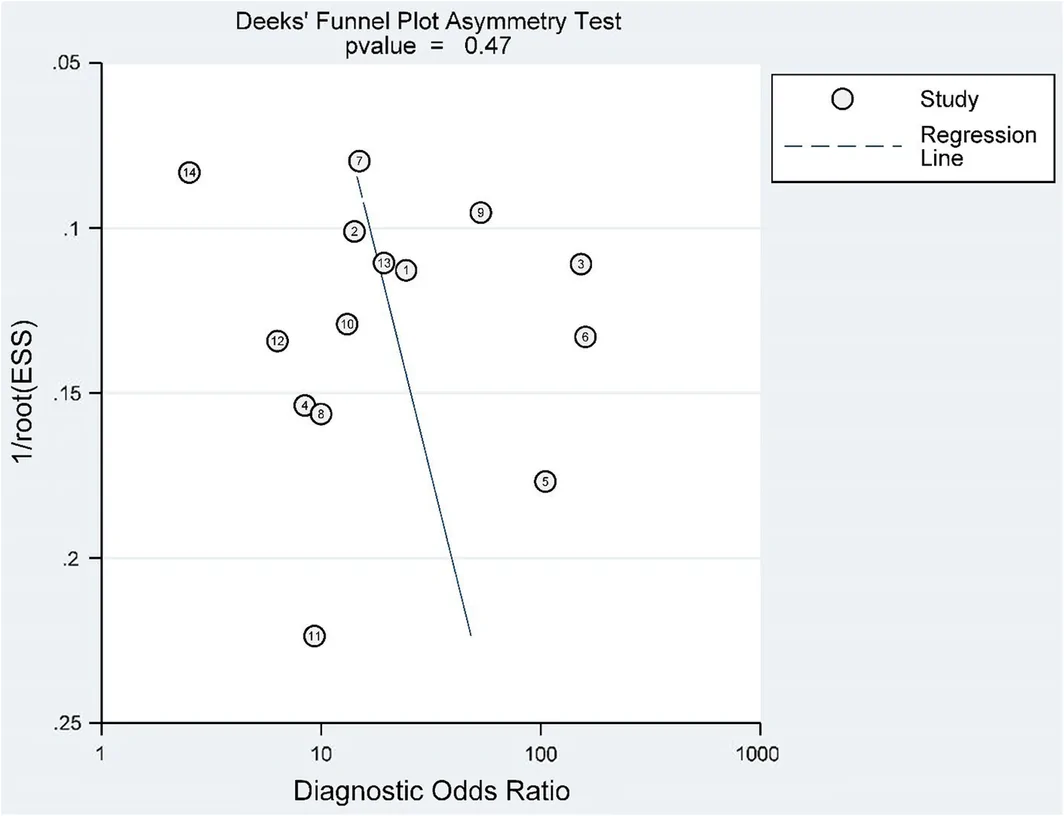
Deeks plot of meta‐analysis of salivary biomarkers used to diagnose breast cancer.

Despite the guidance provided by Cochrane review, we require further research to improve our understanding of the determinants and extent of publication bias in test accuracy studies. In this regard, it is necessary to evaluate a single test as well as compare tests.^3^

Lastly, the suggestion to exclude distant metastases, combined primary breast cancers, and cancer in other organs from the study is interesting. However, we would like to note that an attractive concept does not lead to the desired acceptable result. This is because exclusion of such confounders would rely on the details provided in the articles and this information was not available. Therefore, even though we appreciate your suggestion, it was practically impossible to do it.

## AUTHOR CONTRIBUTIONS

**Maryam Koopaie:** Writing – original draft (equal); writing – review and editing (equal). **Mahnaz Fatahzadeh:** Writing – original draft (equal); writing – review and editing (equal). **Sajad Kolahdouz:** Writing – original draft (equal); writing – review and editing (equal).

## Supporting information


Data S1.

## Data Availability

Data sharing is not applicable to this article as no new data were created or analyzed in this study.
